# Case report: Combination therapy with selinexor, decitabine and half-dose CAG regimen for relapsed elderly acute myeloid leukemia

**DOI:** 10.3389/fonc.2024.1391329

**Published:** 2024-09-16

**Authors:** Xueya Zhang, Yuqi Sun, Jinfa Zhong

**Affiliations:** Department of Hematology, The Second Affiliated Hospital of Fujian Medical University, Quanzhou, Fujian, China

**Keywords:** elderly acute myeloid leukemia, relapsed, selinexor, decitabine, half-dose CAG regimen

## Abstract

The treatment of elderly patients diagnosed with acute myeloid leukemia (AML) poses significant challenges. Currently, one promising strategy in therapeutic interventions for geriatric individuals revolves around the utilization of small molecule targeted drugs either independently or in conjunction with demethylating agents. In this report, we present the successful attainment of complete remission in an elderly female patient with relapsed AML, the patient underwent treatment with a combination of Selinexor, decitabine, and a half-dose CAG regimen for two cycles. Subsequently, the patient has sustained this remission through consolidation therapy involving a medium dose of Ara-c. This therapeutic regimen has demonstrated favorable outcomes in the management of relapsed AML in elderly individuals. Furthermore, the adverse reactions were manageable. In order to devise an efficacious treatment regimen for elderly patients suffering from relapsed and refractory acute myeloid leukemia, it is imperative to incorporate a larger cohort of cases for clinical investigation.

## Introduction

The treatment of elderly patients diagnosed with acute myeloid leukemia (AML) poses significant challenges for hematologists due to the presence of strong heterogeneity, poor performance status, multiple comorbidities, and adverse prognostic factors (1). However, in recent years, the introduction and utilization of diverse novel drugs have led to substantial advancements in the management of AML in the elderly population. According to data analysis conducted by the MD Anderson Cancer Center (2), the response rate to intensive chemotherapy in elderly patients with AML is estimated to be between 40% and 50%. However, these patients face a high early mortality rate of 26% to 36% within 1 to 2 months, and their median survival is limited to only 4 to 6 months. Furthermore, the overall one-year survival rate for elderly AML patients is less than 30%, which is significantly lower compared to the response rate of 70% to 80% and the long-term survival rate of 40% to 50% observed in younger patients.

Currently, one promising strategy in therapeutic interventions for geriatric individuals revolves around the utilization of small molecule targeted drugs either independently or in conjunction with demethylating agents. In the case of elderly patients experiencing relapsed and refractory AML, it is advisable to consider participation in clinical trials, explore novel drug therapies, and implement optimal supportive treatment approaches (1, 2). In this report, we present the successful attainment of complete remission in an elderly female patient with relapsed AML, achieved through the administration of a half-dose CAG regimen in combination with selinexor and decitabine.

## Case presentation

**Figure 1:**
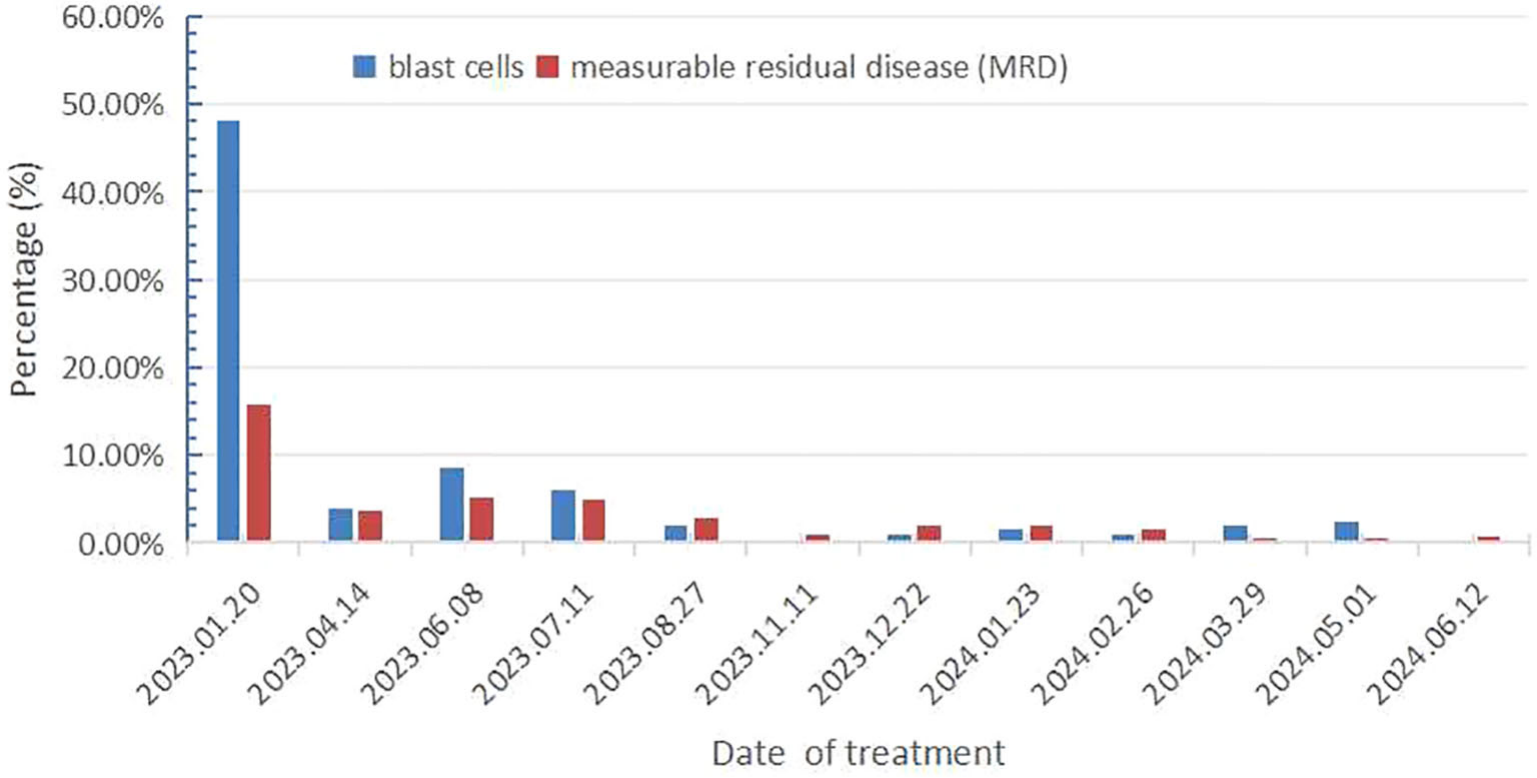
A schematic figure illustrating the treatment course from diagnosis to remission.

**Figure 2:**
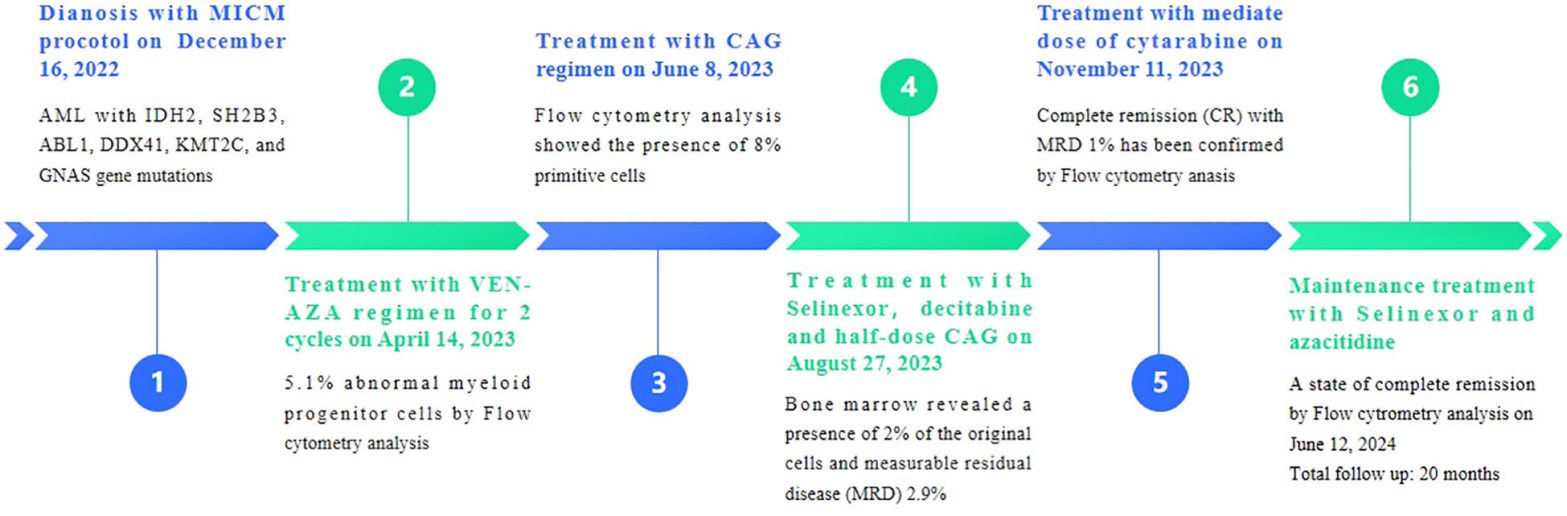
Results of bone marrow primitive cell count and flow cytometry immunophenotyping for measurable residual disease.

On December 16, 2022, a 71-year-old female patient was admitted to our hospital presenting with abdominal distension lasting for a duration of one week. The patient’s medical history did not reveal any other concurrent illnesses. The complete blood count analysis revealed a white blood cell count (WBC) of 1.7 × 10^9^/L, a hemoglobin level of 94 g/L, and a platelet count of 43 × 10^9^/L. Subsequent bone marrow aspiration revealed AML with 58.5% myeloblasts. Flow cytometry analysis demonstrated that blast cells accounted for 48.1% of the sample, exhibiting positive expression of CD13, CD33, CD34, CD38dim, CD105, CD117, HLA-DR, CD45, and MPO. Additionally, the next-generation sequencing results indicated the presence of IDH2 (c.419G>A p.R140Q), SH2B3 (c.527_528del p.V176Afs*6), ABL1 (c.1826_1828del p.K609del), DDX41 (c.1574G>A p.R525H), KMT2C (c.1173C>A p.C391*), and GNAS (c.608T>C p.L203P) gene mutations. The conventional cytogenetic analysis revealed a karyotype of 46, XX (20). A treatment regimen consisting of azacitidine (AZA: 119mg on days 1-5) and venetoclax (VEN: 20mg on day 1, 40mg on day 2, 80mg on day 3, and 100mg on days 4-28) and voriconazole prophylaxis was given with one cycle. Subsequent bone marrow analysis indicated a 5% of residual blast cells, suggesting complete remission with incomplete hematological recovery (CRi). On April 14, 2023, bone marrow aspiration revealed that approximately 4% of the cells were blasts, while immature monocytes accounted for approximately 1%. Additionally, flow cytometry immunophenotyping identified 3.6% of myeloid progenitor cells with abnormal phenotypes. Following this, on April 14, 2023, AZA+VEN targeted therapy was administered, consisting of AZA 120mg subcutaneous injection on days 1-5 and VEN 100mg oral administration on days 1-28, in combination with antifungal prophylaxis with voriconazole 200mg oral administration every 12 hours. A subsequent review of bone marrow aspiration on May 25, 2023, indicated that blast cells accounted for 8.5% of the total cell population. Flow cytometry analysis revealed the presence of 5.1% abnormal myeloid progenitor cells, indicating an early recurrence after achieving CRi. Consequently, the patient was initiated on the CAG regimen chemotherapy on June 8, 2023. This regimen included the administration of 20mg intravenous bolus injection daily for four days of aclarubicin, 16mg subcutaneous injection every 12 hours for 14 days of cytarabine, and 300ug subcutaneous injection for 14 days of granulocyte colony-stimulating factor (G-CSF). A subsequent review of the bone marrow on July 7, 2023, revealed the presence of 8% blast cells. Considering the patient’s unsatisfactory response to first line chemotherapy, we decided to adopt the XPO1 inhibitor selinexor given its promising activity (see Discussion) in combination with decitabine and half dose CAG regimen on July 11, 2023. This consisted of decitabine 25mg administered on days 1 to 5, cytarabine 16mg every 12 hours on days 3 to 10, aclarubicin 20mg on days 3 and 4, G-CSF 300ug on days 3 to 10, and selinexor 60mg once a week for four weeks. On August 25, 2023, a re-examination of the bone marrow revealed a presence of 2% of the original cells and measurable residual disease (MRD) 2.9% by flow cytometry. Commencing from August 27, 2023, the patient was administered selinexor at a dosage of 60mg once a week for four weeks, along with decitabine (25mg d1-5) and a half dose of CAG (aclarubicin 20mg d3-4, cytarabine 16mg q12h d3-9, G-CSF 300ug d3-9). On September 19, 2023, another bone marrow re-examination was conducted, indicating a persistence of 2% cells and MRD of 1%. A subsequent review of the bone marrow on November 9, 2023, confirmed complete remission (CR) with MRD 1%. The patient then underwent a regimen of consolidation chemotherapy consisting of a intermediate dose of cytarabine (Ara-c) (1.5g/d q12h) for three days in one cycle, starting on November 11, 2023. Up to date, the patient was treated with maintenance selinexor + AZA after Ara-c and her bone marrow examination reflects a state of CR (**Figures 1**, **2**). Last date of follow up was July 20, 2024 and overall survival of the patient so far reached 20 months.

## Discussion

The efficacy of intensive chemotherapy in elderly patients with acute myeloid leukemia (AML) is frequently suboptimal due to factors such as drug resistance, compromised performance status, multiple organ dysfunction, and significant treatment-related toxicities, resulting in elevated early mortality rates (1). Consequently, there is a growing need to explore alternative therapeutic approaches for this patient population. One such approach involves the utilization of novel agents and treatment regimens. Notably, the combination of venetoclax (VEN) and azacitidine (AZA) has emerged as a standard regimen for AML patients aged ≥ 75 years or those deemed unsuitable for intensive chemotherapy (3, 4). Despite showing initial promise, the issue of resistance to combination therapy poses a significant challenge, leading to disappointing outcomes for patients with relapsed/refractory acute myeloid leukemia (R/R AML).

A patient’s Eastern Cooperative Oncology Group performance status greater than 2 rendered our patient unsuitable for intensive chemotherapy. Consequently, she was administered induction chemotherapy utilizing the AZA and VEN regimen. Due to the intricate biological nature of this disease and the variations in initial treatment approaches, it is imperative to prioritize approved therapies that specifically target the R/R AML subgroup. Consequently, conducting clinical trials to treat this subgroup of patients is one of the effective strategies For the majority of patients, allogeneic hematopoietic cell transplantation (HCT) stands as the sole potential curative treatment strategy. Prior to HCT, a common practice involves administering a combination of chemotherapy and targeted therapy as a rescue treatment to alleviate the burden of leukemia (1, 2).

The CAG regimen, which consists of cytarabine, aclarubicin, and granulocyte colony-stimulating factor, has been extensively utilized in China and Japan for treating both newly diagnosed and R/R AML. It has shown good tolerance and minimal cardiotoxicity (5). A meta-analysis of CAG revealed a complete remission rate of 60.1% in R/R AML patients, indicating its potential to overcome AML resistance (6). A previous study reported a complete remission rate of 46.5% in R/R AML patients treated with the CAG regimen. Furthermore, the efficacy and safety of CAG in the treatment of AML patients who did not respond to initial induction chemotherapy was demonstrated (7). These findings suggest that the CAG regimen holds promise as a potential therapeutic option for both newly diagnosed and R/R AML cases.

Selinexor, an inhibitor of exportin-1 (XPO-1), effectively promotes nuclear retention and functional activation of tumor suppressor proteins, thereby inducing apoptosis in cancer cells (8). The prevalent overexpression of XPO-1 in various tumors, including AML, underscores the significance of developing novel therapeutic approaches, especially for relapsed AML cases (9). Given the poor prognosis associated with relapse in 10-60% of AML patients, the demand for new treatment strategies is particularly pressing. After a promising phase I trial (10), A phase II study was conducted using a combination of selinexor, cytarabine, and idarubicin in patients diagnosed with R/R AML (11). A total of forty-two patients, with a median age of 59.5 years, were enrolled in the study. However, due to the occurrence of prolonged aplasia and a high incidence of febrile neutropenia (85%) and grade 3/4 diarrhea (56%), the initial selinexor dosage of 40 mg/m^2^ administered twice weekly for a duration of 4 weeks was subsequently reduced to 60 mg twice weekly for a period of 3 weeks. This adjustment resulted in a notable decrease in the occurrence of febrile neutropenia (33%) and severe diarrhea (40%). The overall response rate observed in this study was 50% (8).

Based on above studies and given her frailty, the patient underwent treatment with a combination of Selinexor, decitabine, and a half-dose CAG regimen for two cycles, resulting in complete remission. Subsequently, the patient has sustained this remission through consolidation therapy involving a medium dose of Ara-c. This therapeutic regimen has demonstrated favorable outcomes in the management of relapsed AML in elderly individuals. As side effect, the patient exhibited bone marrow suppression and pancytopenia, which subsequently resolved through symptomatic intervention, leading to a restoration of normal hematopoiesis within two weeks. Furthermore, the adverse reactions were manageable. In order to devise an efficacious treatment regimen for elderly patients suffering from R/R AML, it is imperative to incorporate a larger cohort of cases for clinical investigation.

## Data Availability

The original contributions presented in the study are included in the article/**Supplementary Materials**, further inquiries can be directed to the corresponding author.
