# Supplementary figures and images for: Case report: Combination therapy with selinexor, decitabine and half-dose CAG regimen for relapsed elderly acute myeloid leukemia
Source: Front Oncol. 2024 Sep 16;14:1391329. doi: 10.3389/fonc.2024.1391329 (PMC11439872; doi:10.3389/fonc.2024.1391329)

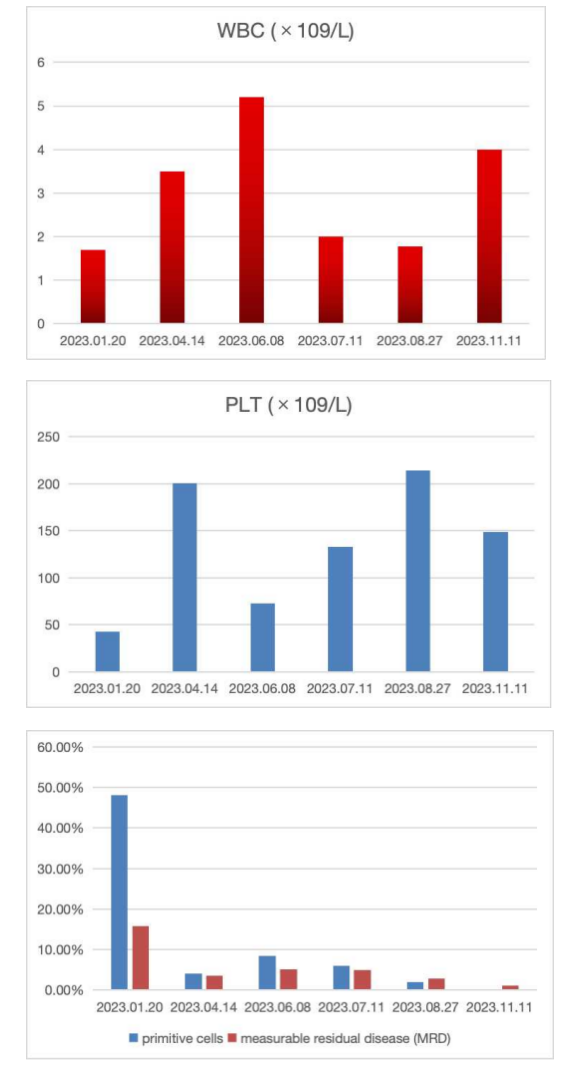

Supplement: Supplementary file 1 [file Image1.png]
